# The Mutual Interplay between Bone, Glucose and Lipid Metabolism: The Role of Vitamin D and PTH

**DOI:** 10.3390/nu15132998

**Published:** 2023-06-30

**Authors:** Vittoria Carmela Danese, Jessica Pepe, Federica Ferrone, Luciano Colangelo, Viviana De Martino, Luciano Nieddu, Giancarlo Ferrazza, Enrico Panzini, Roberto Pascone, Frank Blocki, Salvatore Minisola, Cristiana Cipriani

**Affiliations:** 1 Department of Clinical, Internal Medicine, Cardiovascular and Anesthesiological Sciences, Sapienza University of Rome, Viale del Policlinico 155, 00161 Rome, Italy; vitto_danese@yahoo.it (V.C.D.); jessica.pepe@uniroma1.it (J.P.); fede89ferrone@gmail.com (F.F.); luciano.colangelo@uniroma1.it (L.C.); viviana.demartino@uniroma1.it (V.D.M.); 2 Faculty of Economics, Università degli Studi Internazionali di Roma, Via Cristoforo Colombo 200, 00147 Rome, Italy; l.nieddu@gmail.com; 3 Department of Immunohematology and Transfusion Medicine, Policlinico Umberto I, Sapienza University of Rome, Viale del Policlinico 155, 00161 Rome, Italy; g.ferrazza@policlinicoumberto1.it (G.F.); e.panzini@policlinicoumberto1.it (E.P.); 4 Department of Pediatrics, Sapienza University of Rome, Viale Regina Elena 324, 00161 Rome, Italy; roberto.pascone@fondazione.uniroma1.it; 5 DiaSorin Inc., 1951 Northwestern Avenue, Stillwater, MN 55082, USA; cin9lan7tsaf@outlook.com

**Keywords:** vitamin D, PTH, glucose, cholesterol, LDL, HDL, triglycerides, lipid

## Abstract

Background. We sought to investigate the mutual interplay between bone, glucose and lipid metabolism in a wide cohort of community-based subjects. Methods. We studied 1240 blood donors (F/M ratio 1/3.2, mean age 41.9 ± 11.7 SD). Serum ionized (Ca^++^), magnesium (Mg^++^), 25-hydroxy-vitamin D [25(OH)D], PTH-1-84, 1,25-dihydroxyvitamin D [1,25(OH)_2_D], total cholesterol (C), HDL-C, triglycerides and glucose were measured and LDL-C levels were calculated in all subjects. Results. 25(OH)D negatively correlated with BMI (R = −0.11), PTH (R = −0.16) (*p* < 0.0001), total C (R = −0.06, *p* < 0.05) and triglycerides (R = −0.13, *p* < 0.0001) and positively with 1,25(OH)_2_D (R = 0.12) and creatinine (R = 0.17) (*p* < 0.0001). Serum PTH positively correlated with total C (R = 0.08, *p* < 0.01), LDL-C (R = 0.1, *p* < 0.001), triglycerides (R = 0.09, *p* < 0.01) and glucose (R = 0.15, *p* < 0.0001) and negatively with HDL-C (R = −0.09, *p* < 0.01). The odds of showing abnormal serum triglycerides and HDL-C increased as 25(OH)D decreased (*p* < 0.0001 and *p* < 0.03) and PTH increased (*p* < 0.03 and *p* = 0.05), while the odds of showing abnormal LDL-C levels increased in association with elevated PTH (*p* < 0.01). Conclusion. Vitamin D, PTH, glucose and lipid metabolism are mutually influenced. Hypovitaminosis D predisposes toward worsening lipid profiles through the actions of PTH, while serum PTH levels per se associate with higher glucose and LDL-C levels.

## 1. Introduction

Bone is a key regulator of several metabolic pathways involved in a number of physiologic and pathophysiological conditions. Beyond mineral metabolism, glucose and adipose tissue homeostasis are the two most studied endocrine systems for which regulation as a determinant role of the skeleton has been extensively demonstrated [1,2]. More specifically, pre-clinical and clinical evidence has shown that a mutual interplay exists between these three systems [1,2]. As a consequence, the perturbation of one of the three may be associated with pathological conditions in the other two. Clinical examples of such complex metabolic pathways may be found in patients with type 2 diabetes and/or obesity, in which increased skeletal fragility, high prevalence of hypovitaminosis D and secondary hyperparathyroidism are common conditions [2]. Additional clinical studies have demonstrated that hypovitaminosis D represents a risk factor for metabolic syndrome and dyslipidemia. Lee et al. performed a meta-analysis of 19 cross-sectional and 4 cohort studies which disclosed a linear dose–response association between metabolic syndrome and serum 25-hydroxy-vitamin D [25(OH)D] concentrations [3]. A recent systematic review and meta-analysis of 57 observational and 2 cohort studies from 21 different countries reported an 18% reduction in the odds of dyslipidemia in association with higher serum 25(OH)D levels; in particular, hypertriglyceridemia and low HDL cholesterol (C) levels demonstrated a non-linear association with lower 25(OH)D levels [4].

With reference to the parathyroid hormone (PTH), emerging clinical evidence suggests a direct association between the serum levels of the hormone, body weight and body mass index (BMI), as illustrated by studies in patients with primary hyperparathyroidism (PHPT), wherein a higher prevalence of metabolic syndrome is described [5,6,7]. Interestingly, a recent study by Yuan et al. showed that these metabolic effects are seen for modest, incident increases in PTH, given that the relationships between this hormone, body weight and visceral adipose tissue are only significant in PHPT patients in the first tertile of PTH levels (21.4–65.8 pg/mL) [7]. Conversely, PTH was negatively correlated with all the parameters associated with the adipose tissue in the highest tertile (147.0–2511.7 pg/mL), thus showing an inverted U-shape curve in its relationship with body weight, BMI and visceral area [7]. As far as lipid metabolism, an atherogenic profile is described in patients with PHPT [6]. Lower serum HDL-C, elevated triglycerides, very-low-density lipoprotein (VLDL) and LDL-C levels are described in both hypercalcemic and normocalcemic PHPT [5,6]. Moreover, worst lipid profiles were observed in PHPT patients with serum PTH levels within the normal range [8]. In healthy subjects, a few studies investigated the effect of PTH on adipose tissue metabolism. In a Swedish cohort of 1016 healthy individuals aged 70 years and older, a significant positive association was observed between serum PTH, waist circumference, BMI, systolic and diastolic blood pressure, serum triglycerides and Homeostatic Model Assessment of Insulin Resistance (HOMA-IR) and a negative one with HDL-C [9].

To date, there have been few studies assessing the mutual interplay between bone, glucose and lipid metabolism in healthy subjects of different ages. Moreover, the relative influence and role of different factors involved in bone metabolism have not been detailed. We conducted an observational study with the primary aim of investigating this specific topic in a community-based cohort of healthy women and men of a wide age range.

## 2. Materials and Methods

This research complies with the World Medical Associations Declaration of Helsinki (Ethical Principles for Medical Research involving Human Subjects). This study was approved by Policlinico Umberto I, Sapienza University of Rome Ethics Committee; all subjects provided written informed consent.

We studied 1240 healthy subjects (949 men and 291 women, 63 postmenopausal) aged 18–68 years (mean ± SD 41.9 ± 11.7) recruited among voluntary blood donors of the Policlinico Umberto I, Sapienza University of Rome (latitude 41°54′39″24 N) from March 2014 to July 2015. The cohort represents a subgroup of participants from an ongoing project aimed at evaluating the prevalence and natural history of normocalcemic primary hyperparathyroidism who agreed to participate in the present sub-study [10]. 

Medical history was assessed and physical examination as well as laboratory exams (blood count, liver and renal function, coagulation, etc.) were performed in all subjects to exclude medical conditions that could represent contraindications to donation. Subjects taking drugs that affect glucose or lipid metabolism and mineral metabolism other than calcium and/or vitamin D (i.e., thiazides, bone active agents) were excluded from analysis. Height and weight were measured in all fasting subjects with light clothing and no shoes by stadiometer to the nearest 0.001 m for body mass index (BMI) calculation, and with a calibrated bathroom scale with precision up to 0.1 kg, respectively.

Fasting blood samples were collected between 8.00 and 10.00 a.m. for measurement of serum ionized calcium (Ca^++^), ionized magnesium (Mg^2+^), 25(OH)D, 1,25-dihydroxyvitamin D [1,25(OH)_2_D], parathyroid hormone 1–84 (PTH), serum total and HDL-C, triglycerides and plasma glucose. Ca^2+^ and Mg^2+^ were measured within 2 h and plasma glucose within 30 min of collection, on 4 °C room temperature; blood samples were stored at −80 °C and assayed in one batch at the end of the study for determination of other analytes. Serum Ca^++^ and Mg^++^ were measured by biochemical analyzer NOVA 8 (Nova Biomedical, Waltham, MA, USA); serum 25(OH)D and PTH by chemiluminescence-immunoassay (LIAISON^®^, DiaSorin USA, Stillwater, MN, USA) and 1,25(OH)_2_D by LIAISON XL^®^; serum total C and HDL-C, triglycerides and plasma glucose were by enzymatic test with the Cobas c 311 analyzer (Cobas^®^); serum LDL-C was calculated by the Friedewald formula: LDL cholesterol = total C-HDL-C- (triglycerides/5). As detailed in a previous paper from our group [10], the Vitamin D TOTAL Assay (DiaSorin USA, Stillwater, MN, USA) with a measurement range of 4–150 ng/mL and functional sensitivity ≤4.0 ng/mL was employed; intra- and inter-assay precision were 8.9% and 12.8%, respectively, with 100% detection of both 25-hydroxyvitamin D_2_ and 25-hydroxyvitamin D_3_. We used the PTH-1-84 assay (DiaSorin USA, Stillwater, MN, USA) with 100% specificity, 4.1% intra- and 5.2% inter-assay coefficients of variation, and the 1,25 Dihydroxyvitamin D (DiaSorin USA, Stillwater, MN, USA) with measurement range of 5–200 pg/mL and functional sensitivity ≤ 5.0 pg/mL, 100% specificity to 1,25(OH)_2_D_3_ and 1,25(OH)_2_D_2_ without cross-reactivity to other forms of vitamin D. The intra- and inter-assay coefficients of variation were 2.1% and 4.9%, respectively.

### Statistical Analysis

Results are presented as mean values ± SD. Associations between demographic, anthropometric and laboratory data were tested by Spearman rank-order correlation in all subjects; the same analyses were assessed in subjects with vitamin D insufficiency (defined as serum 25(OH)D levels < 20 ng/mL) [11] with the aim of assessing the association between hypovitaminosis D and glucose and lipid profile. Similarly, the association analyses were made in subjects with PTH levels above the upper limit of the range considered as normal (36.6 pg/mL) to test the hypothesis of any influence of elevated serum PTH levels on glucose and lipid metabolism. 

We applied a logistic model using serum 25(OH)D and PTH as covariates to study their effect on the odds of showing abnormal levels of serum total C, LDL-C, HDL-C, triglycerides and plasma glucose. Abnormal serum levels were defined as follows: total C > 200 mg/dL, LDL-C > 116 mg/dL, HDL-C < 50 mg/dL in women and <40 mg/dL in men; triglycerides > 150 mg/dL; glucose > 100 mg/dL [12,13,14]. Significant *p* values < 0.05 were considered and the R package version 3.5.0 (R Core Team, 2018) was used for analysis.

## 3. Results

Table 1 shows demographics, anthropometric characteristics and laboratory findings in all subjects. As shown, mean serum 25(OH)D levels were at the lower limit of the range considered as optimal in the general population [11], while mean serum levels of other analytes were within the normal range.

**Figure 1:**
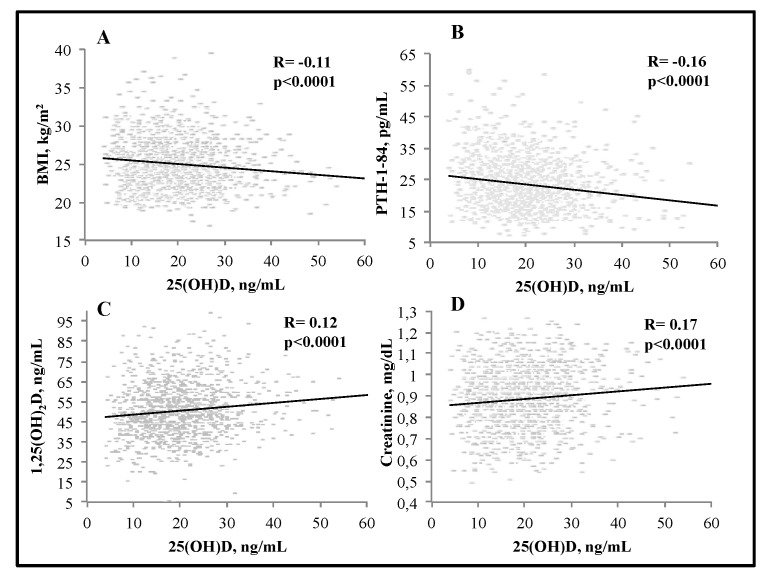
Associations between serum 25(OH)D levels and BMI (**A**), serum PTH (**B**), 1,25(OH)_2_D (**C**) and creatinine (**D**); assessed by Spearman rank-order correlation.

**Figure 2:**
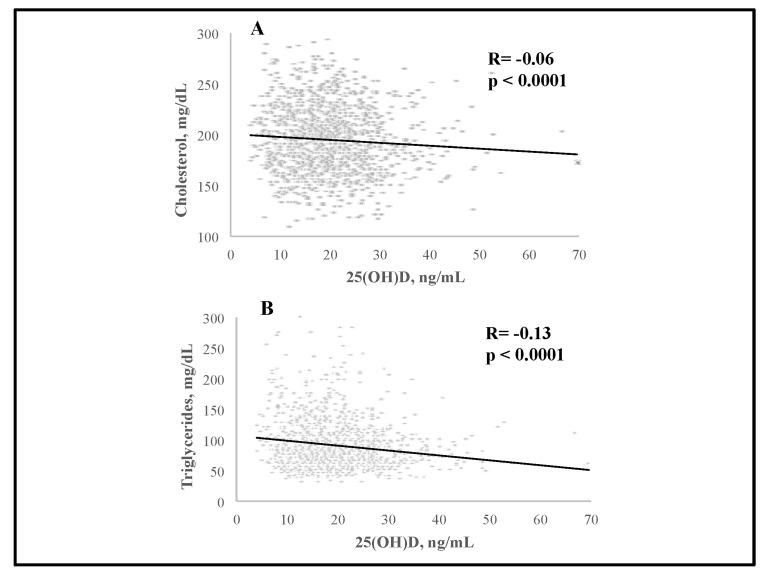
Associations between serum 25(OH)D levels and total cholesterol (**A**) and triglycerides (**B**); assessed by Spearman rank-order correlation.

**Figure 3:**
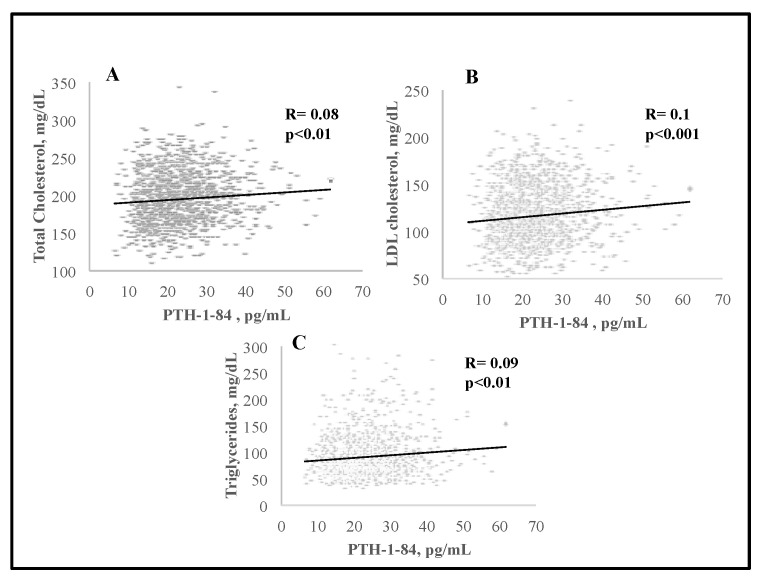
Associations between serum PTH levels and total (**A**), LDL cholesterol (**B**) and triglycerides (**C**); assessed by Spearman rank-order correlation.

**Figure 4:**
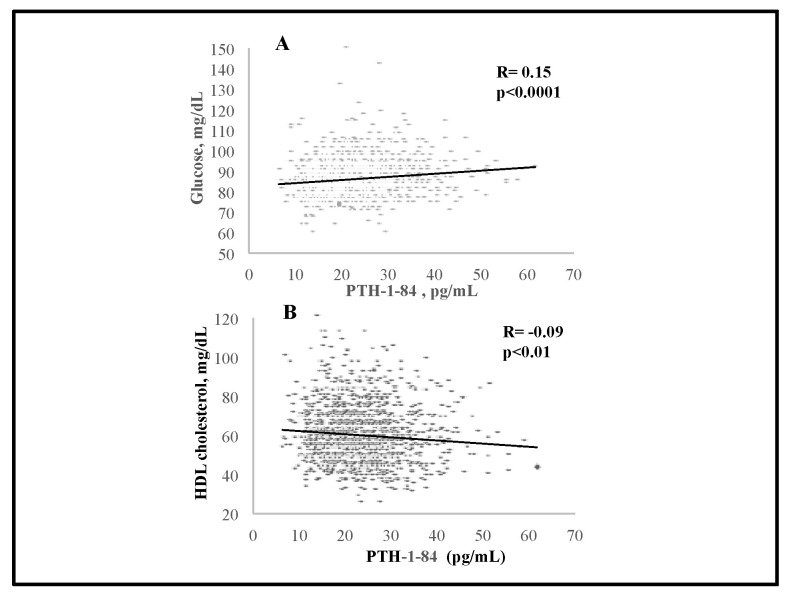
Associations between serum PTH levels and HDL cholesterol (**A**) and glucose (**B**); assessed by Spearman rank-order correlation.

We observed significant negative associations between serum 25(OH)D and BMI (R = −0.11, *p* < 0.0001), and PTH (R = −0.16, *p* < 0.0001); serum 25(OH)D levels were positively associated with serum 1,25(OH)_2_D (R = 0.12) and creatinine (R = 0.17) (*p* < 0.0001 for both) (Figure 1). There was a significant negative association between serum 25(OH)D and total C (R = −0.06, *p* < 0.05) as well as triglycerides (R = −0.13, *p* < 0.0001) (Figure 2). Interestingly, serum PTH positively correlated with total C (R = 0.08, *p* < 0.01), LDL-C (R = 0.1, *p* < 0.001), triglycerides (R = 0.09, *p* < 0.01) and glucose (R = 0.15, *p* < 0.0001) (Figure 3 and Figure 4). Conversely, serum PTH was negatively associated with HDL-C (R = −0.09, *p* < 0.01) (Figure 4). No association between serum Ca^++^ and Mg^++^ levels and parameters of glucose and lipid metabolism were observed.

**Figure 5:**
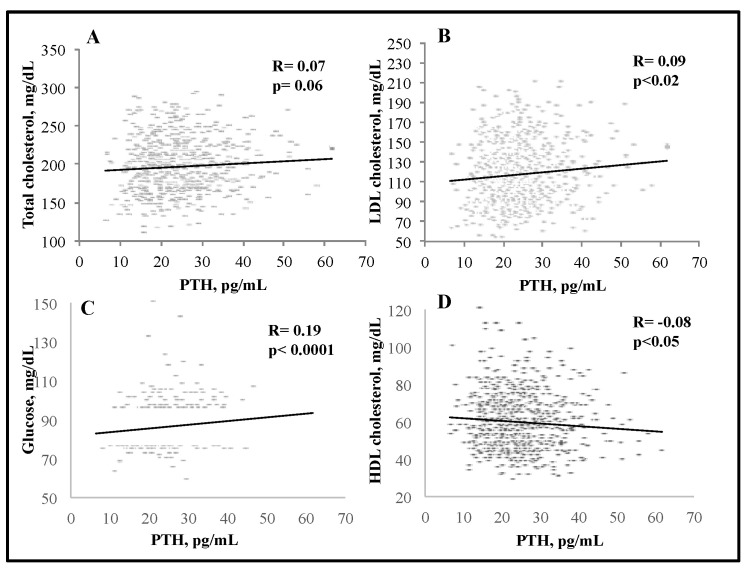
Associations between serum PTH levels and total (**A**), LDL (**B**) and HDL cholesterol (**C**) and glucose (**D**) in the subgroup of subjects whose serum 25(OH)D levels were <20 ng/mL; assessed by Spearman rank-order correlation.

In the subgroup of subjects with serum 25(OH)D < 20 ng/mL (*n* = 670), there was no association between serum 25(OH)D and parameters of glucose and lipid metabolism. Conversely, the associations between serum PTH, glucose and lipid metabolism were substantially maintained. A positive association between serum PTH levels and total C (R = 0.07, *p* = 0.06), LDL-C (R = 0.09, *p* < 0.02) and glucose (R = 0.19, *p* < 0.0001) was observed, while HDL-C negatively correlated with PTH (R = −0.08, *p* < 0.05) (Figure 5).

The analysis of the subgroup of subjects with elevated serum PTH levels (>36.6 pg/mL, *n* = 85) showed no significant association between serum PTH and any of the parameters of glucose and lipid metabolism. Interestingly, serum PTH positively correlated with total C (R = 0.08, *p* < 0.01) and LDL-C (R = 0.11, *p* < 0.001), triglycerides (R = 0.08, *p* < 0.01), glucose (R = 0.14, *p* < 0.0001), and negatively with HDL-C (R = −0.09, *p* < 0.01) in subjects whose PTH was <36.6 pg/mL (*n* = 1155).

The logistic model demonstrated that the odds ratio of showing abnormal serum triglycerides and HDL-C levels increased as 25(OH)D decreased (HDL-C: *p* < 0.03; triglycerides: *p* < 0.0001) and PTH increased (triglycerides: *p* < 0.03; HDL-C: *p* = 0.05) (Table 2). The analysis was marginally significant as far as total C (*p* = 0.06 for both decrease in 25(OH)D and increase in PTH). The odds of showing abnormal values of LDL-C increased only in association with increases in serum PTH levels (*p* < 0.01); similar results were observed with abnormal glucose levels, albeit here, the significance was marginal (*p* = 0.09) (Table 2).

## 4. Discussion

There is a significant and mutual relationship between the vitamin D-PTH axis, glucose and lipid metabolism. Specifically, hypovitaminosis D, defined as serum 25(OH)D levels < 20 ng/mL, according to the consensus in the general population [11], predisposes to abnormal lipid profiles through the action of PTH. Similar to their action on bone metabolism, the well-known inverse relationship between serum 25(OH)D and PTH corroborated in our cohort seems to play a synergistic and significant role in influencing plasma glucose and serum lipid levels.

Due to its involvement in many metabolic pathways, vitamin D deficiency may have several metabolic consequences that potentially translate into pathologic conditions of various target organs [15]. A number of observational and longitudinal studies performed in wide cohorts across different geographical areas have associated poor vitamin D status with many extra-skeletal issues [15]. In this context, controversies have risen from some of the most recent randomized controlled trials (RCTs) that failed to find a superiority of vitamin D supplementation compared to placebo in exerting beneficial effects on multiple extra-skeletal endpoints [16,17]. Similar arguments have been applied to the effect of vitamin D on glucose and lipid metabolism.

The pathophysiology of the multiple actions of vitamin D beyond those on bone and mineral metabolism focus on the expression by a number of systems and organs of vitamin D receptor (VDR), 1α-hydroxylase (the enzyme mediating the activation of the hormone) and the intra-nuclear response to vitamin D by many genes [15]. As such, vitamin D exerts its favorable effects on glucose metabolism by a fine regulation of insulin sensitivity involving different organs (liver, adipose tissue, skeletal muscle and kidneys) [2,18]. Actions of vitamin D include the stimulation of the expression of glucose transporters, hormones and signaling pathways enhancing insulin sensitivity, increased metabolism of fatty acids and the suppression of the renin–angiotensin–aldosterone system [18]. These mechanisms are amongst those considered as playing a central role in the complex and multifactorial link between vitamin D deficiency and metabolic syndrome [18,19]. As far as lipid metabolism, vitamin D may exert direct (by inhibiting the sterol regulatory element-binding proteins with the consequent suppression of free fatty acid synthesis and uptake) and indirect positive effects (by ameliorating insulin sensitivity, enhancing intestinal calcium absorption that in turn reduces hepatic secretion of triglycerides) [4]. Consistent with these mechanisms of vitamin D action on insulin and adipose tissue metabolism, our observations of a negative correlation between serum 25(OH)D, BMI, total C and triglycerides confirm data from previous observational studies [3,4,20]. Meta-analyses of cross-sectional and cohort studies calculated a 15–20% and 4% reduced risk of metabolic syndrome and dyslipidemia, respectively, in association with 10 ng/mL increases in serum 25(OH)D [3,4,20]. Bahadorpour et al. reported 7% and 3% marginally significant risk reductions in hypertriglyceridemia and low HDL-C, respectively, for similar 10 ng/mL increases in serum 25(OH)D levels [4]. In two meta-analyses using random effect models to account for differences across studies in the method of 25(OH)D determination, Rafiq et al. demonstrated an inverse relationship between serum 25(OH)D and fasting glucose and insulin levels in diabetic and non-diabetic subjects [21,22]. In our cohort, plasma glucose was only associated with serum PTH levels in subjects with hypovitaminosis D. Hence, in terms of mechanisms, our study provides intriguing data on the link between hypovitaminosis D and altered glucose and lipid profile, suggesting a key role of PTH.

The suppression of serum PTH secretion by parathyroid glands is one of the most important indirect mechanisms through which vitamin D may regulate insulin, glucose and adipose tissue metabolism, with significant effect on serum lipids as well [4,18]. Similar to vitamin D, PTH may also exert a significant action on the adipose tissue. Through the increase in intracellular calcium levels, elevated PTH may suppress catecholamine-mediated lipolysis and stimulate fatty acid synthesis and release [4,7,9]. Interestingly, we observed an increased risk of the typical metabolic syndrome-associated lipid profile (abnormal serum triglycerides and HDL-C) in association with both decreased serum 25(OH)D and increased PTH levels, consistently with the aforementioned pathophysiological mechanisms, as well as with evidence from some intervention studies [23,24]. Several clinical trials have disclosed the efficacy of vitamin D in reducing serum PTH with concomitant beneficial effects on cardiovascular risk factors (lipids, fasting glucose, insulin, HOMA-IR, blood pressure) in subjects with different metabolic disorders (overweight/obesity, non-alcoholic fatty liver disease, diabetes) [24]. Notwithstanding, results have been inconsistent across other studies. The meta-analysis of four studies focusing on the effect of vitamin D supplementation in patients with coronary artery disease, while reporting a significant decrease in serum PTH during the 2–6 month follow-up period, did not reveal any change in serum triglycerides, LDL and HDL-C in vitamin D compared with the placebo group [25]. Additionally, Qi et al. recently performed a systematic review and meta-analysis of 13 RCTs with a composite of 1,076 subjects with metabolic syndrome and reported no effects of vitamin D supplementation on either lipid profile or serum PTH levels [26]. Conversely, a systematic review and meta-analysis of 81 RCTs involving roughly 10,000 subjects aged 18–85 years demonstrated that vitamin D supplementation significantly ameliorates lipid profile (triglycerides, LDL-C and HDL-C serum levels) while reducing serum PTH [23]. Healthy, community-dwelling as well as institutionalized subjects and patients with several chronic conditions (diabetes, chronic kidney disease, etc.) were included in the meta-analysis. In pre-diabetics, the benefits from vitamin D supplementation are inconsistently reported by meta-analyses of RCTs due to the heterogeneity of these studies [27,28,29]. Yu et al. described a reduction only in 2 h glucose levels after oral glucose testing, while Zhang et al. observed an amelioration in fasting glucose and insulin levels as well as hemoglobin A1c (HbA1c) in association with vitamin D supplementation [27,28]. Finally, a recent meta-analysis of individual participant data from three RCTs showed a 15% risk reduction in diabetes and a 30% regression to normal plasma glucose levels in subjects with pre-diabetes [29].

In the aggregate, some but not all of the evidence from intervention studies is in line with data from ours and other observational studies, as well as the pathophysiology describing the positive actions of vitamin D on glucose and lipids. The treatment of vitamin D deficiency may benefit healthy subjects and some classes of high-risk patients in terms of amelioration of some outcomes associated with glucose and lipid metabolism, while no effects are seen in patients on secondary prevention and in those with metabolic syndrome, in which presumably other mechanisms of lipid metabolism regulation play a prominent role [23,24,25,26]. Inconsistent results across studies may also be evaluated in relation with major differences in the target population and in baseline serum 25(OH)D levels, heterogeneity of follow-up and of vitamin D supplementation, with various consequent effects on serum 25(OH)D levels and on its potential of suppressing serum PTH. As Bahadorpour et al. pointed out, to date, the relevant number of studies and meta-analyses on the topic have been insufficient in defining a target serum 25(OH)D level that is associated with obtaining and maintaining optimal glucose and lipid profiles [4]; accordingly, the magnitude of serum PTH reduction with similar favorable effects is far from being known.

As demonstrated in both hypercalcemic and normocalcemic PHPT, mild-to-moderate PTH increases may be associated with higher serum levels of fasting glucose, insulin, HOMA-IR, total and LDL-C and triglycerides; concomitant with normalization in serum PTH after surgery, a significant reduction in glucose and lipid parameters was observed [6,30,31]. Conversely, patients with more severe PHPT and higher serum PTH levels are more prone to weight loss independent of serum calcium, creatinine and nutritional status [7,32,33]. The recently described mechanisms of PTH action on the adipose tissue may explain these findings. He et al. reported the potential of high concentrations of PTH in favoring the browning of the white adipose tissue in a murine model of PHPT; higher percentages of brown adipose tissue assessed by ^18^F-fluorodeoxyglucose-positron emission tomography/computed tomography (^18^F-FDG-PET/CT) images were described in PHPT as compared to control subjects as well [33]. The observations made in our cohort are consistent with this fascinating evidence, as only serum PTH levels within the normal range were associated with altered glucose and lipid profile, while higher levels were not.

The limitations of our study are associated with its cross-sectional nature. We have no longitudinal data that would be of help in best defining the relative role of vitamin D and PTH in influencing glucose and lipid levels according to factors such as season, use of vitamin D and/or calcium supplements and eventual changes in nutritional status (weight loss/gain, dietary habits, etc.). Additionally, some parameters (e.g., Fibroblast Growth Factor 23, FGF23, sclerostin, insulin), for which significant roles in the relationship between bone and glucose metabolism are described, were not assessed. Notwithstanding, this is one of the few reports on this specific topic involving a wide cohort of different ages in which the influence of confounding factors (primarily drugs and comorbidities) was excluded. As intervention studies performed so far have demonstrated, the clinical relevance of our results may be applied to high-risk subjects, as well as to the general population [16], while recognizing the benefit of adherence to a more standardized and comprehensive methodology. In particular, the inclusion of subjects with baseline vitamin D deficiency may enhance the quality of the results from RCTs performed in this research area.

In conclusion, the observations made in our study confirm that vitamin D, PTH, glucose and lipid metabolism are mutually influenced. Abnormal lipid profiles may be seen in the setting of hypovitaminosis D in association with serum PTH, whose levels independently correlate with higher serum LDL-C levels and plasma glucose. Future studies in different pathological conditions will clarify the clinical relevance of these data and mechanisms underlying such associations.

## Figures and Tables

**Table 1:** Demographic, anthropometric characteristics and laboratory findings (mean ±SD) in all subjects.

Parameter	All Subjects (*n* = 1240)	Normal Range
Age, years	41.9 ± 11.7	-
Female/male ratio	1/3.2	-
Men, *n*	949	
Pre-menopausal women, *n*	228	
Post-menopausal women, *n*	63	
Weight, kg	76.7 ± 13	-
Height, cm	175 ± 8	-
BMI, kg/m^2^	24.9 ± 3.3	18.5–25
	19.9 ± 8.4	>20
PTH, pg/mL	23.5 ± 8.3	6.5–36.6
1,25(OH)_2_D, pg/mL	50.5 ± 13.2	19.9–79.3
Ca^++^, mmol/L	1.29 ± 0.03	1.17–1.33
Mg^++^, mmol/L	0.53 ± 0.05	0.45–0.6
Creatinine, mg/dL	0.89 ± 0.15	0.7–1.2
Total cholesterol, mg/dL	195 ± 34.2	80–232
LDL cholesterol, mg/dL	116.4 ± 32	69–118
HDL cholesterol, mg/dL	60 ± 15.2	44–64
Triglycerides, mg/dL	91 ± 46.5	45–163
Glucose, mg/dL	86.2 ± 9.5	68–99

**Table 2:** Results from the logistic model assessing the effect of serum 25(OH)D and PTH on the odds ratio (OR) of showing abnormal lipid and glucose levels.

Parameter	25(OH)D	PTH
Total cholesterol	OR 0.99 (95% CI 0.97–1) *p* = 0.06	OR 1 (95% CI 0.99–1.03) *p* = 0.06
LDL cholesterol	OR 0.99 (95% CI 0.98–1.01) *p* = NS	OR 1 (95% CI 1.01–1.04) *p* < 0.01
HDL cholesterol	OR 0.97 (95% CI 0.94–0.99) *p* < 0.03	OR 1 (95% CI 0.99–1.04) *p* = 0.05
Triglycerides	OR 0.94 (95% CI 0.91–0.96) *p* < 0.0001	OR 1.02 (95% CI 1–1.05) *p* < 0.03
Glucose	OR 1 (95% CI 0.97–1.02) *p* = NS	OR 1 (95% CI 0.99–1.05) *p* = 0.09

NS: not significant.

## Data Availability

Some or all datasets generated during the current study are not publicly available but are available from the corresponding author upon reasonable request.
